# Hospital admissions due to diseases of arteries and veins peaked at physiological equivalent temperature −10 to 10 °C in Germany in 2009–2011

**DOI:** 10.1007/s11356-015-5791-x

**Published:** 2015-12-03

**Authors:** Ivy Shiue, David R. Perkins, Nick Bearman

**Affiliations:** Faculty of Health and Life Sciences, Northumbria University, Newcastle upon Tyne, NE1 8ST England UK; Owens Institute of Behavioral Research, University of Georgia, Athens, USA; Center for Climate Change Communication, George Mason University, Fairfax, USA; School of Environmental Sciences, University of Liverpool, Liverpool, UK

**Keywords:** Hospital admission, Weather, Biometeorology, Artery, Vein, Thrombosis, Atherosclerosis

## Abstract

We aimed to understand relationships of the weather as biometeorological and hospital admissions due to diseases of arteries and veins by subtypes, which have been scarcely studied, in a national setting in recent years. This is an ecological study. Ten percent of daily hospital admissions from the included hospitals (*n* = 1,618) across Germany that were available between 1 January 2009 and 31 December 2011 (*n* = 5,235,600) were extracted from Statistisches Bundesamt, Germany. We identified I70-I79 Diseases of arteries, arterioles and capillaries and I80-I89 Diseases of veins, lymphatic vessels and lymph nodes by International Classification of Diseases version 10 as the study outcomes. Daily weather data from 64 weather stations that covered 13 German states including air temperature, humidity, wind speed, cloud cover, radiation flux and vapour pressure were obtained and generated into physiologically equivalent temperature (PET). Two-way fractional-polynomial prediction was plotted with 95 % confidence intervals. For most of the subtypes from diseases of arteries and veins, hospital admissions slightly peaked in spring and dropped when PET was at 10 °C. There were no other large differences across 12 months. Admissions of peripheral vascular diseases, arterial embolism and thrombosis, phlebitis and thrombophlebitis, oesophageal varices and nonspecific lymphadenitis peaked when PET was between 0 and −10 °C, while others peaked when PET was between 0 and 10 °C. More medical resources could have been needed on days when PETs were at −10 to 10 °C than on other days. Adaptation to such weather change for health professionals and the general public would seem to be imperative.

**Electronic supplementary material:**

The online version of this article (doi:10.1007/s11356-015-5791-x) contains supplementary material, which is available to authorized users.

## Introduction

### Evidence before this study

Environmental factors have been central to many human chronic diseases, and the weather is no exception. The effect of the weather has been noted in scientific literature since the 1930s as increased hospital admissions due to coronary occlusion and heart failure were observed in correlation with low temperature that has prompted the concern on the influence of the seasonality effect (Bean and Mills 1938). The hypothesised mechanism was that an acute change in environmental temperature, being too cold or too hot (Bhaskaran et al. 2009). tends to increase myocardial oxygen consumption and may induce cardiac arrhythmias or an anginal attack (Ansari and Burch 1969; Epstein et al. 1969; Milo-Cotter et al. 2006). In addition, activation of the sympathetic nervous system and secretion of catecholamine could increase in response to low temperature that could be observed by the increased heart rate and peripheral vascular resistance (Hanna 1999).

### Knowledge gap

However, overall across the globe, conflicting results on the effect of the weather on human health outcomes have been presented in the literature. While there are complex interactions between the weather and human health outcomes that have been observed, methodological concerns on the risk assessment from previous research have been brought up recently (Modesti 2013). Seemingly, correlating air temperature and human health outcomes might not be adequate since all the climatic variables usually interacted with each other at the same time. There has also been some difference observed between air temperature and the weather as biometeorological incorporating the relevant meteorological parameters (Shiue and Matzarakis 2011). Recently, we have already reported that hospital admissions of hypertension, angina, myocardial infarction and ischemic heart disease peaked at physiologically equivalent temperature (PET) 0 °C in Germany in 2009–2011 (Shiue et al. 2015). However, such data and examination on other clinical outcomes such as diseases of arteries and veins by subtypes were scarcely studied and documented.

### Study aim

Therefore, following this context, we aimed to firstly investigate the monthly variations of hospital admissions due to diseases of arteries and veins by subtypes and then to correlate with the weather in a national setting in recent years.

## Methods

### Study sample

This is an ecological study. Daily hospital admissions with all diagnoses including emergency admissions in the same study period were extracted from the database held in Statistisches Bundesamt (more details via https://www.destatis.de/EN/Homepage.html), Wiesbaden, Germany. Statistisches Bundesamt randomly selects 10 % of hospital admissions from each German hospital at the end of each year and stores the data for the research purpose. Currently, there are 1,618 hospitals in their record list. The admission (‘primary diagnosis’) is coded using the International Classification of Diseases version 10 codes (WHO 2009). In this study, we identified hospital admissions due to I70-I79 Diseases of arteries, arterioles and capillaries and I80-I89 Diseases of veins, lymphatic vessels and lymph nodes by subtypes as the study outcomes that were with enough number of events to be examined statistically and to protect any individual from being identified due to small numbers. Daily historical meteorological data including air temperature, humidity, wind speed, radiation flux, cloud cover and vapour pressure between 1 January 2009 and 31 December 2011 (three full calendar years) were obtained from the Federal Ministry of Transport, Building, and Urban Development (more details via http://www.dwd.de/). This time period was selected because of the limited availability of quality data on hospital admissions.

### Variables and analyses

In the first step, we used a Geographic Information System to map out the included weather stations (64 out of 78 representative stations across Germany according to meteorologists at the Federal Ministry of Transport, Building, and Urban Development and based on the completeness of meteorological data) from each state of Germany (see Supplementary Fig. 1). There are 16 states in total. In the second step, for each weather station, we generated all the included meteorological parameters mentioned above into a single index called PET on a daily basis as the study exposure. We then averaged daily PETs from each weather station within the same state into monthly values.

PET, with a widely known unit degrees Celsius, has been known to be used to consider a heat balance of the human body under the standard conditions in an outdoor setting and initially created to characterise and evaluate the human bioclimate in a physiological setting (Höppe 1999). PET takes into consideration thermoregulatory processes such as sweat rate and blood vessel dilation and allows the user of the model to predict thermal attributes of the body such as sweat rate, core temperature and skin temperature. Calculating PET requires atmospheric, geographical and human-physiological inputs. In our case, we used the RayMan software for calculation (more details via http://www.mif.uni-freiburg.de/rayman/intro.htm). Atmospheric inputs include temperature, wind speed, humidity, sky cover and solar radiation. Geographical inputs included altitude and day length (assessed through latitude and longitude). Physiological inputs included gender, age, height, weight, amount of clothing and activity levels (measured in watts). Clothing values were seasonally adjusted for summer, winter and shoulder seasons, and all other physiological inputs are held constant based upon default model parameters. In the current analysis, 64 weather stations from 13 states with complete weather data were included for the statistical analysis. This has excluded three states including Berlin, Saarland and Saxony-Anhalt without any of meteorological parameters that would be needed for PET calculation.

In the third step, we plotted two-way fractional-polynomial prediction with 95 % confidence intervals (CI) in order to better describe the potential non-linear relationships between the weather and hospital admissions with continuous variables presented (more details via http://www.stata.com/manuals13/g-2graphtwowayfpfit.pdf). It is a regression fit plotting process (more details via http://www.stata.com/support/faqs/graphics/gph/graphdocs/twoway-fractional-polynomial-prediction-plot/). For each outcome variable that was included in the present study, two-way fractional-polynomial prediction was performed separately. Due to the German law prohibiting reporting of individual admissions and hospitals, correlational analysis was only made on a monthly basis. From the generated PETs in 2009–2011 (see Supplementary Fig. 2), it was observed that there were no days with heat stress condition (PET >35 °C in Western Europe; Matzarakis and Mayer 1996). Statistical software STATA 12.0 version (STATA, College Station, TX, USA) was used for all the statistical analyses.

### Ethics approval and funding

This study was approved and supported by the EU FP-7 Data without Boundaries project (grant number 262608; more details via http://www.dwbproject.org/) and Statistisches Bundesamt, Germany. Since this study only employed secondary data analyses, no other ethics approval was required.

## Results

**Fig. 1:**
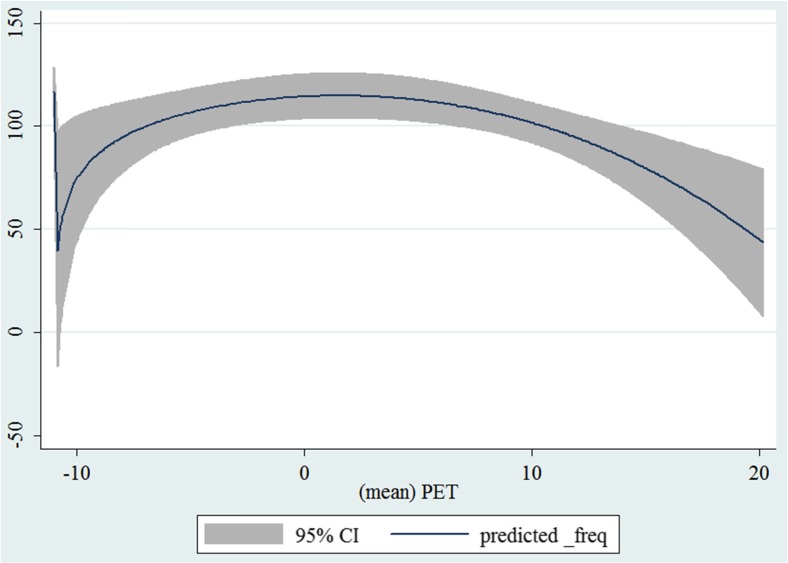
Relationship of PETs and admissions of I70 Atherosclerosis

**Fig. 2:**
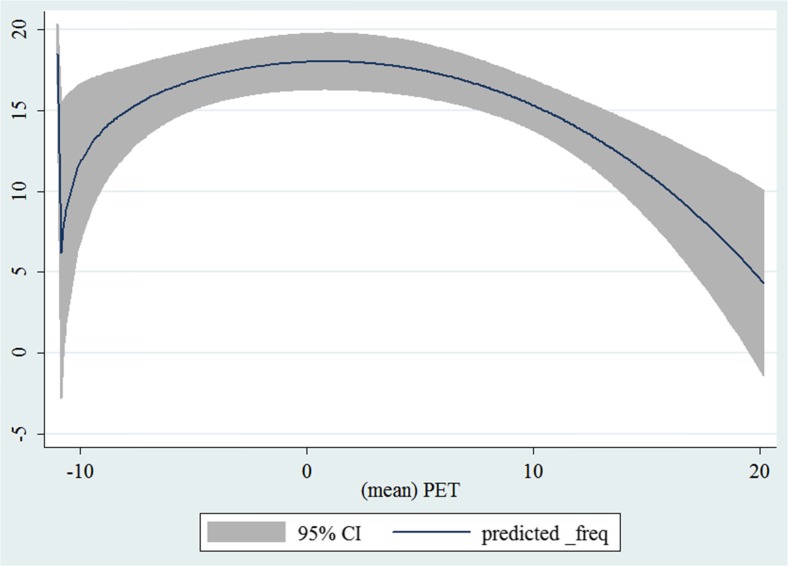
Relationship of PETs and admissions of I71 Aortic aneurysm and dissection

**Fig. 3:**
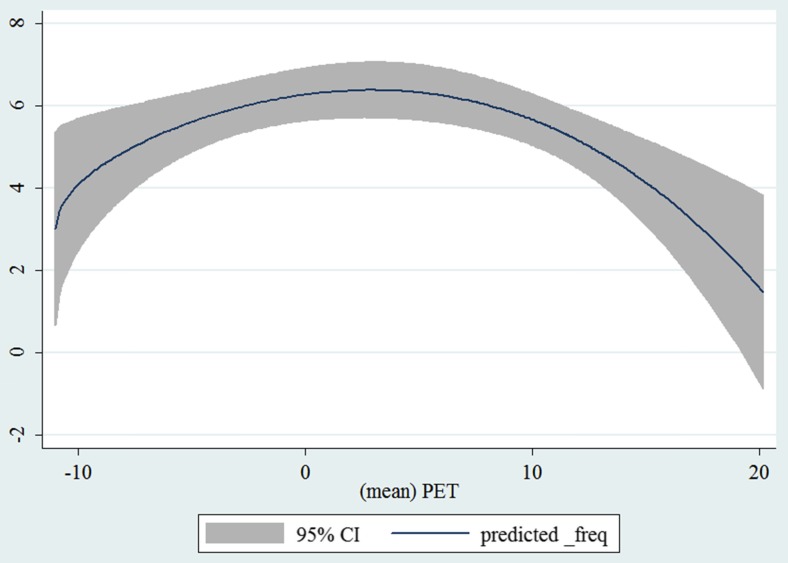
Relationship of PETs and admissions of I72 Other aneurysm and dissection

**Fig. 4:**
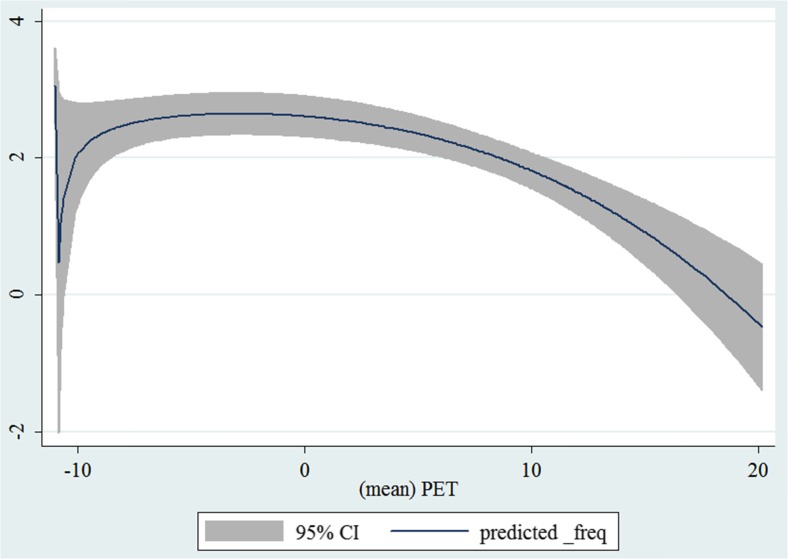
Relationship of PETs and admissions of I73 Other peripheral vascular diseases

**Fig. 5:**
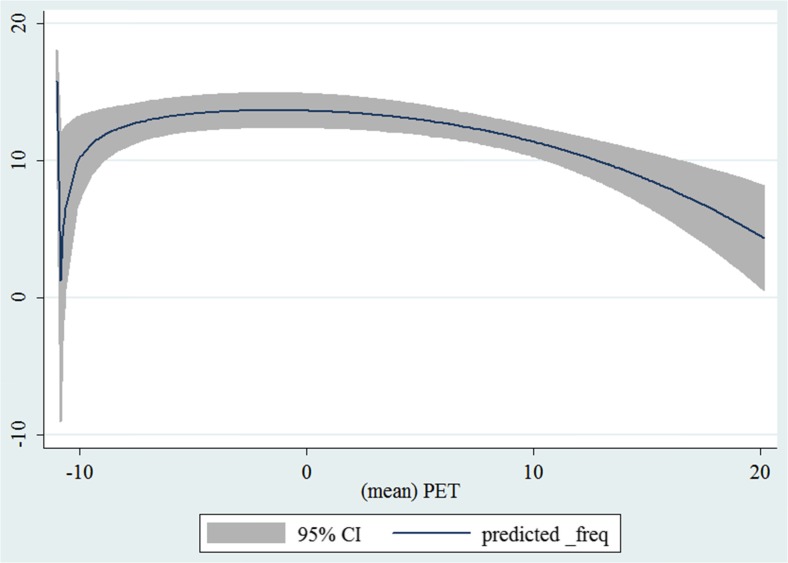
Relationship of PETs and admissions of I74 Arterial embolism and thrombosis

**Fig. 6:**
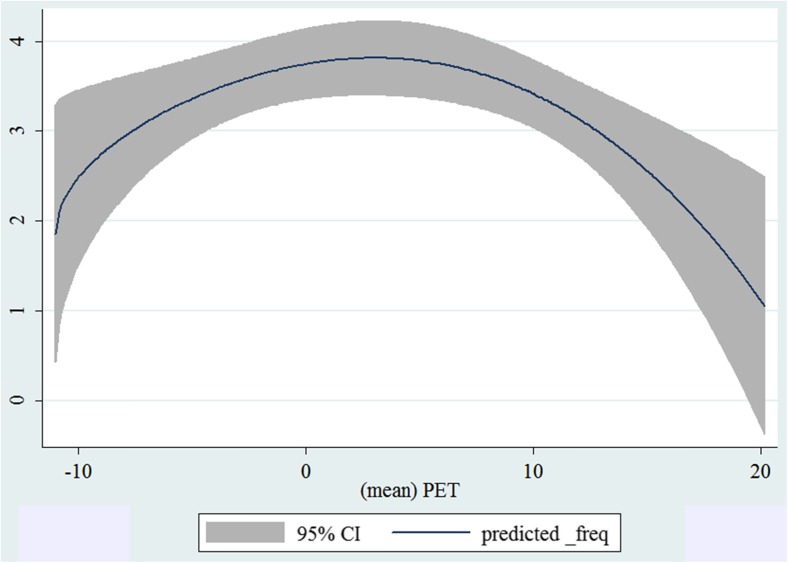
Relationship of PETs and admissions of I77 Other disorders of arteries and arterioles

**Fig. 7:**
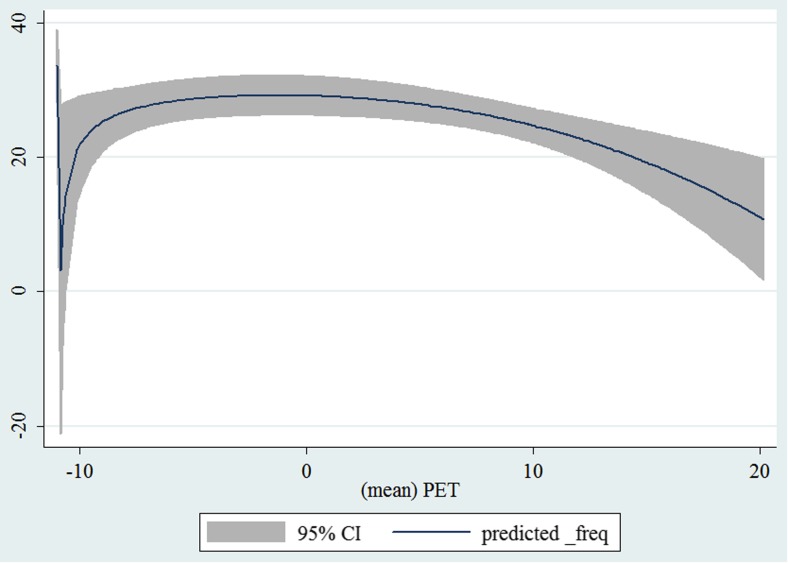
Relationship of PETs and admissions of I80 Phlebitis and thrombophlebitis

**Fig. 8:**
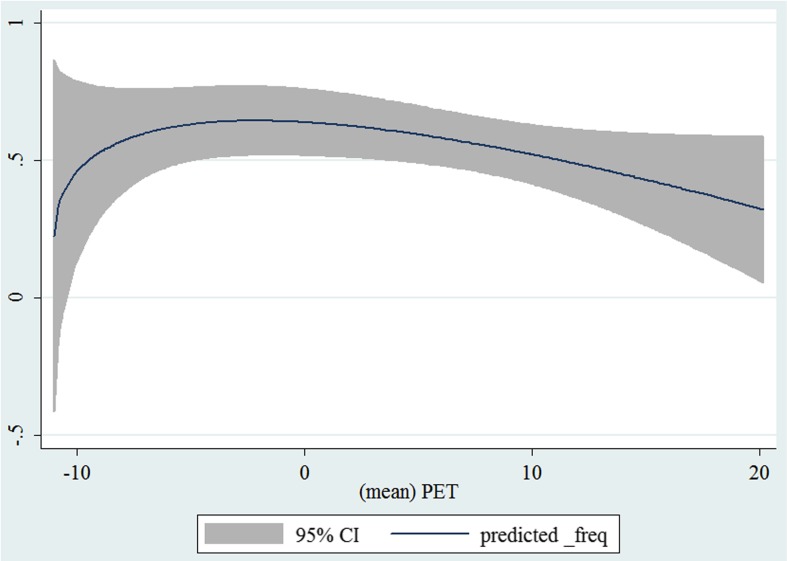
Relationship of PETs and admissions of I81 Portal vein thrombosis

**Fig. 9:**
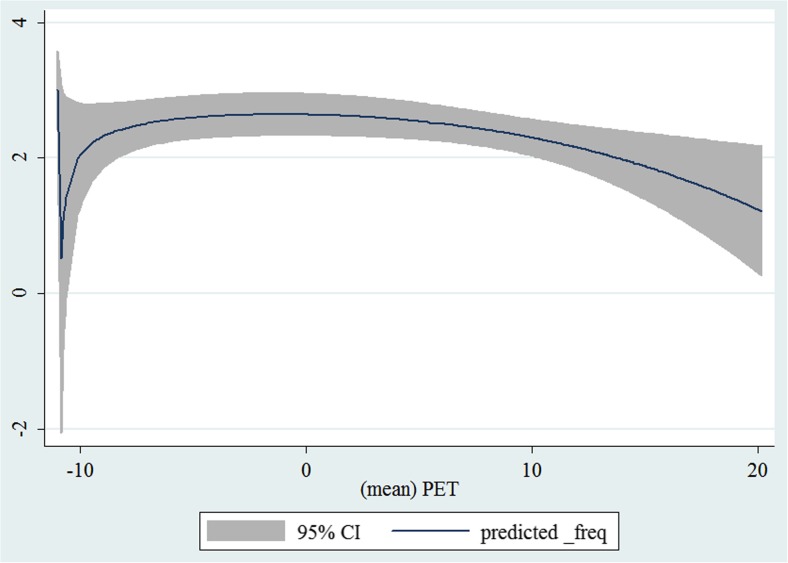
Relationship of PETs and admissions of I82 Other venous embolism and thrombosis

**Fig. 10:**
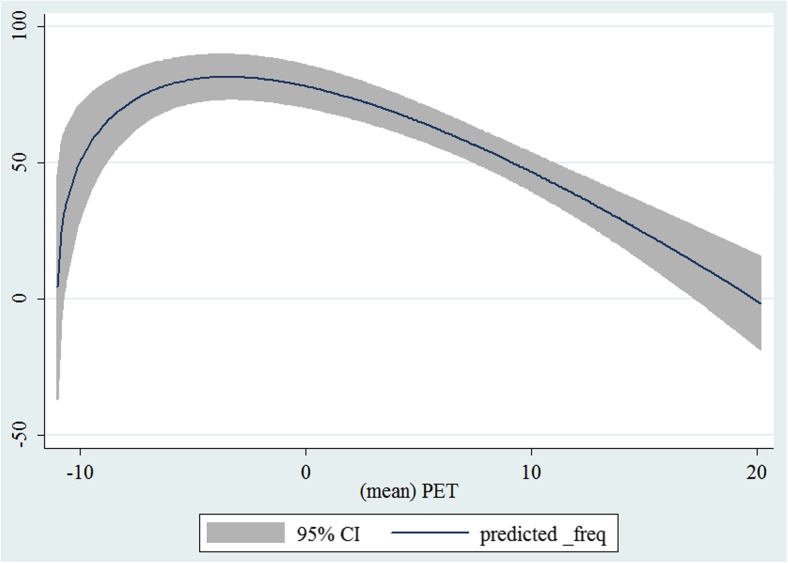
Relationship of PETs and admissions of I83 Varicose veins of lower extremities

**Fig. 11:**
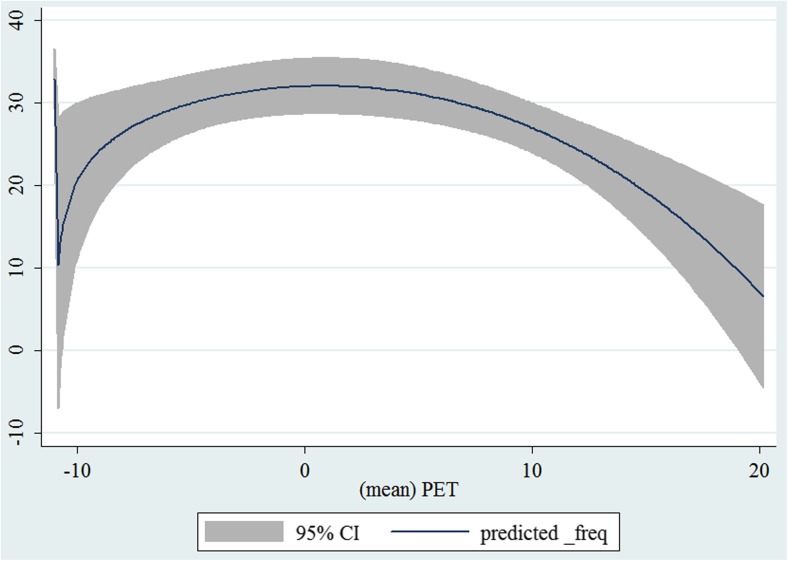
Relationship of PETs and admissions of I84 Haemorrhoids

**Fig. 12:**
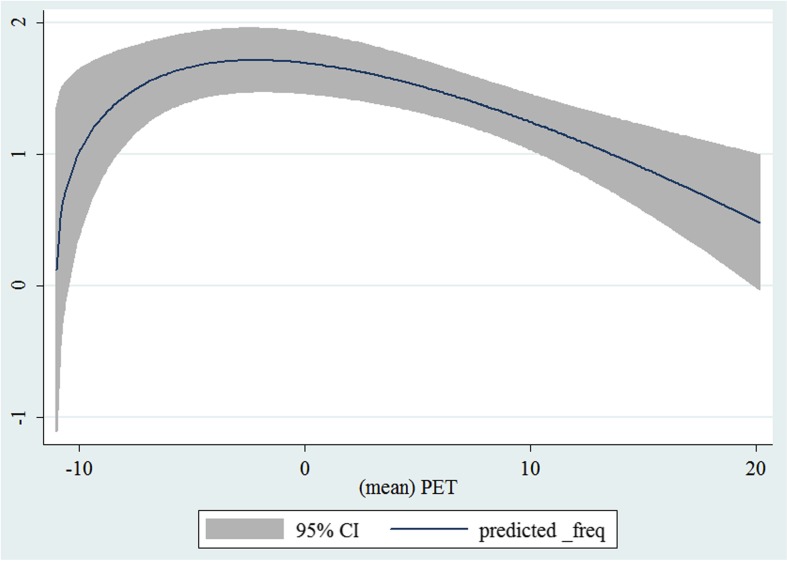
Relationship of PETs and admissions of I85 Oesophageal varices

**Fig. 13:**
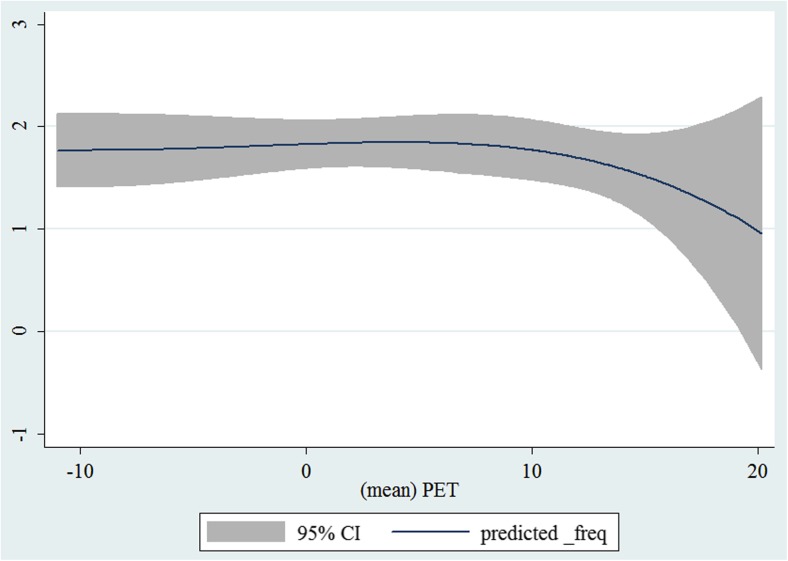
Relationship of PETs and admissions of I86 Varicose veins of other sites

**Fig. 14:**
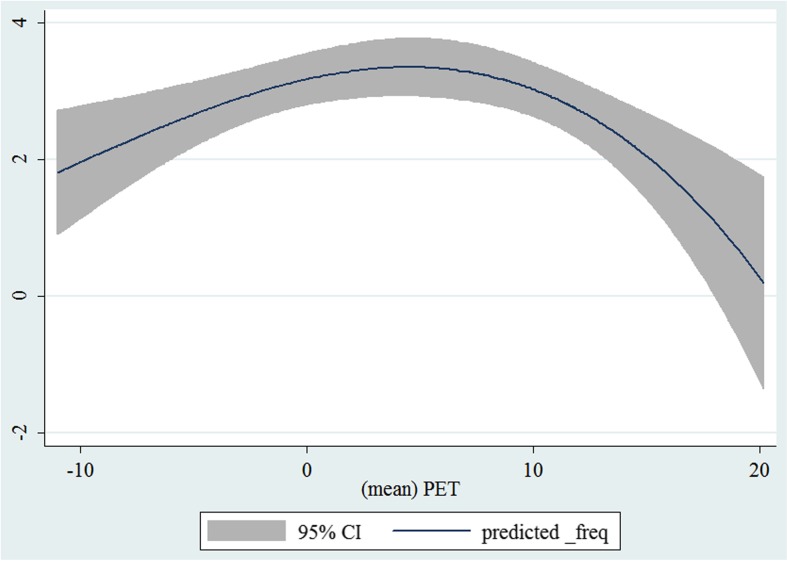
Relationship of PETs and admissions of I87 Other disorders of veins

**Fig. 15:**
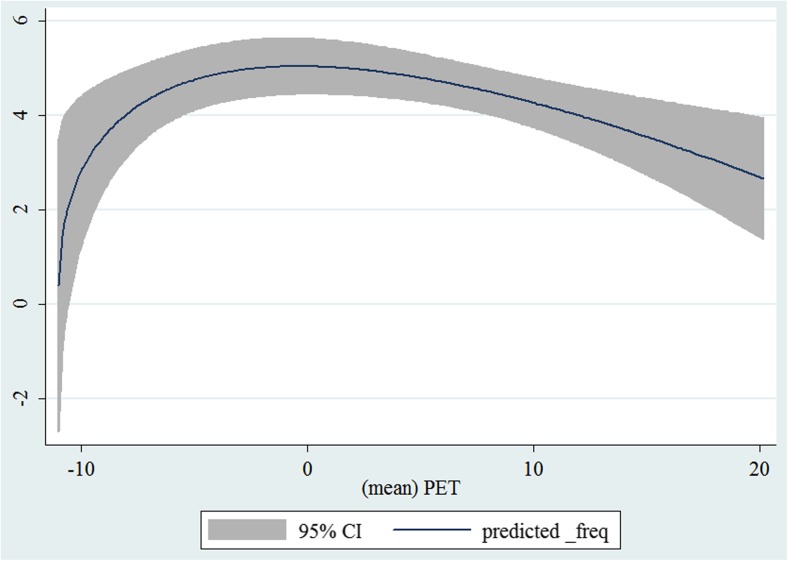
Relationship of PETs and admissions of I88 Nonspecific lymphadenitis

**Fig. 16:**
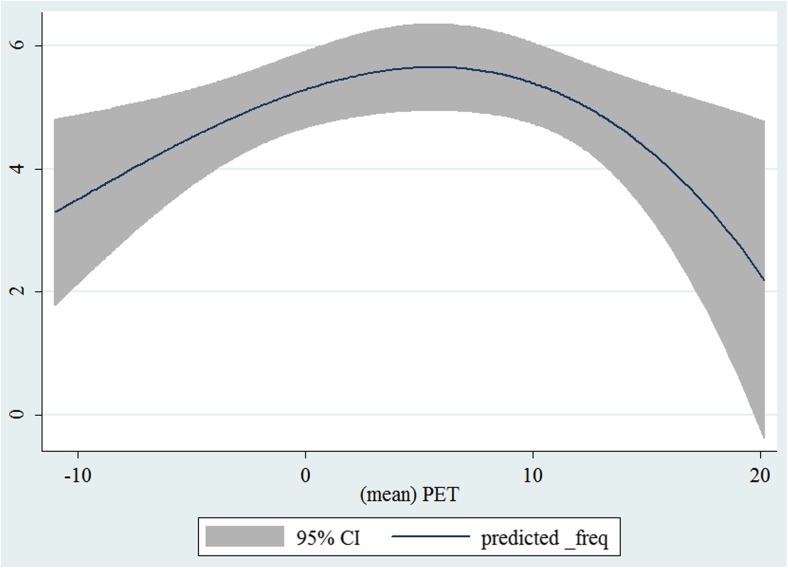
Relationship of PETs and admissions of I89 Other noninfective disorders of lymphatic vessels and lymph nodes

Tables 1 and 2 show monthly number of hospital admissions due to arteries and veins by subtypes. In general, for most of the subtypes from diseases of arteries and veins, hospital admissions slightly peaked in spring and dropped when PET was at 10 °C. There were no other large differences across 12 months. In Figs. 1, 2, 3, 4, 5, 6, 7, 8, 9, 10, 11, 12, 13, 14, 15, and 16, the relationships of PETs and hospital admissions due to arteries and veins by subtypes are displayed accordingly. Admissions of peripheral vascular diseases, arterial embolism and thrombosis, phlebitis and thrombophlebitis, oesophageal varices and nonspecific lymphadenitis peaked when PET was between 0 and −10 °C, while others peaked when PET was between 0 and 10 °C.

**Table 1:** Averaged monthly hospital admissions due to diseases of arteries by subtypes in 2009–2011

	I70 Atherosclerosis	I71 Aortic aneurysm and dissection	I72 Other aneurysm and dissection	I73 Other peripheral vascular diseases	I74 Arterial embolism and thrombosis	I77 Other disorders of arteries and arterioles
January	4,646 (8.7 %)	715 (8.8 %)	231 (8.1 %)	134 (11.9 %)	527 (8.5 %)	137 (8.0 %)
February	4,154 (7.8 %)	647 (8.0 %)	240 (8.4 %)	101 (9.0 %)	567 (9.2 %)	131 (7.6 %)
March	4,868 (9.2 %)	761 (9.4 %)	249 (8.7 %)	126 (11.2 %)	573 (9.3 %)	183 (10.6 %)
April	4,190 (7.9 %)	661 (8.1 %)	235 (8.2 %)	96 (8.5 %)	523 (8.5 %)	143 (8.3 %)
May	4,692 (8.8 %)	685 (8.4 %)	235 (8.2 %)	105 (9.3 %)	548 (8.9 %)	135 (7.8 %)
June	4,545 (8.6 %)	689 (8.5 %)	237 (8.3 %)	70 (6.2 %)	496 (8.0 %)	145 (8.4 %)
July	4,309 (8.1 %)	633 (7.8 %)	246 (8.6 %)	65 (5.8 %)	460 (7.4 %)	147 (8.5 %)
August	4,463 (8.4 %)	647 (8.0 %)	215 (7.5 %)	55 (4.9 %)	478 (7.7 %)	140 (8.1 %)
September	4,570 (8.6 %)	668 (8.2 %)	260 (9.1 %)	77 (6.8 %)	499 (8.1 %)	148 (8.6 %)
October	4,430 (8.3 %)	698 (8.6 %)	255 (8.9 %)	87 (7.7 %)	495 (8.0 %)	140 (8.1 %)
November	4,807 (9.0 %)	722 (8.9 %)	257 (9.0 %)	129 (11.5 %)	526 (8.5 %)	159 (9.2 %)
December	3,497 (6.6 %)	602 (7.4 %)	199 (7.0 %)	82 (7.3 %)	493 (8.0 %)	115 (6.7 %)

**Table 2:** Averaged monthly hospital admissions due to diseases of veins by subtypes in 2009–2011

	I80 Phlebitis and thrombophlebitis	I81 Portal vein thrombosis	I82 Other venous embolism and thrombosis	I83 Varicose veins of lower extremities	I84 Haemorrhoids	I85 Oesophageal varices	I86 Varicose veins of other sites	I87 Other disorders of veins	I88 Nonspecific lymphadenitis	I89 Other noninfective disorders of lymphatic vessels and lymph nodes
January	1,193 (9.1 %)	22 (7.6 %)	96 (7.7 %)	3,022 (10.3 %)	1,194 (8.5 %)	73 (10.3 %)	79 (8.6 %)	121 (8.3 %)	162 (7.3 %)	193 (7.6 %)
February	1,108 (8.4 %)	24 (8.3 %)	109 (8.8 %)	3,080 (10.5 %)	1,216 (8.6 %)	57 (8.1 %)	68 (7.4 %)	124 (8.5 %)	226 (10.2 %)	165 (6.5 %)
March	1,207 (9.2 %)	34 (11.7 %)	118 (9.5 %)	3,405 (11.7 %)	1,439 (10.2 %)	89 (12.6 %)	91 (10.0 %)	127 (8.7 %)	208 (9.4 %)	225 (8.9 %)
April	1,083 (8.2 %)	20 (6.9 %)	91 (7.3 %)	2,490 (8.5 %)	1,158 (8.2 %)	62 (8.8 %)	76 (8.3 %)	118 (8.1 %)	166 (7.5 %)	211 (8.3 %)
May	1,055 (8.0 %)	17 (5.9 %)	105 (8.5 %)	2,105 (7.2 %)	1,150 (8.1 %)	48 (6.8 %)	62 (6.8 %)	138 (9.5 %)	221 (9.9 %)	218 (8.6 %)
June	1,018 (7.7 %)	15 (5.2 %)	109 (8.8 %)	1,670 (5.7 %)	1,188 (8.4 %)	56 (7.9 %)	82 (9.0 %)	130 (9.0 %)	171 (7.7 %)	239 (9.4 %)
July	1,055 (8.0 %)	34 (11.7 %)	102 (8.2 %)	1,241 (4.3 %)	1,132 (8.0 %)	53 (7.5 %)	88 (9.6 %)	113 (7.8 %)	205 (9.2 %)	243 (9.6 %)
August	1,147 (8.7 %)	21 (7.2 %)	110 (8.9 %)	1,390 (4.8 %)	1,134 (8.0 %)	53 (7.5 %)	82 (9.0 %)	117 (8.1 %)	190 (8.5 %)	254 (10.0 %)
September	1,082 (8.2 %)	22 (7.6 %)	99 (8.0 %)	2,236 (7.7 %)	1,119 (7.9 %)	56 (7.9 %)	74 (8.1 %)	127 (8.7 %)	168 (7.6 %)	217 (8.6 %)
October	1,108 (8.4 %)	26 (9.0 %)	103 (8.3 %)	2,964 (10.1 %)	1,124 (8.0 %)	46 (6.5 %)	74 (8.1 %)	127 (8.7 %)	162 (7.3 %)	203 (8.0 %)
November	1,055 (8.0 %)	31 (10.7 %)	97 (7.8 %)	3,481 (11.9 %)	1,325 (9.4 %)	66 (9.3 %)	65 (7.1 %)	124 (8.5 %)	189 (8.5 %)	206 (8.1 %)
December	1,059 (8.0 %)	24 (8.3 %)	102 (8.2 %)	2,146 (7.3 %)	949 (6.7 %)	49 (6.9 %)	73 (8.0 %)	87 (6.0 %)	157 (7.1 %)	165 (6.5 %)

## Discussion

### Weather and atherosclerosis and peripheral vascular diseases

Literature specifically on the relationship of the weather and atherosclerosis admissions is very limited. In animal research (Imai et al. 2006). it was previously observed that cold exposure (4 °C air temperature) in mice significantly reduced serum adiponectin concentrations, which was reportedly involved in protection against atherosclerosis (Okamoto et al. 2002). Literature on the relationship of the weather and admissions due to peripheral vascular diseases is much scarce as well. The present study has therefore provided the evidence using large human sample to support the influence from the weather on these admissions.

### Weather and aortic aneurysm

Literature on the relationship of the weather and aortic aneurysm seems to be more than that on atherosclerosis admissions, although most of the previous studies focused on atmospheric pressure than air temperature. In order to discuss with our current findings, literature on atmospheric pressure solely was not included.

In a single hospital in Homburg/Saar, Germany back in 1994–2011 (Schuld et al. 2013). maximal and average temperature and water vapour pressure were observed significant lower at event days. Similar findings were obtained in Toulouse University Hospital located in France in 1997–2007 (Benouaich et al. 2010). In Blackpool Victoria Hospital located in the UK in 1996–2002, however, no significant associations were found (Repanos and Chadha 2005). In John Radcliffe Hospital also located in the UK in 1991–1995 (Ballaro et al. 1998). a seasonal variation in the incidence of rupture of abdominal aortic aneurysm occurs, with a peak in winter (*p* = 0.003). In two major hospitals in Rome, Italy in 1956–1986 (Sterpetti et al. 1995). admissions due to aortic aneurysm rupture peaked in autumn. In the Clinical Center of Serbia in 1998–2004 (Lasica et al. 2006). the frequency of acute aortic dissection was found to be significantly higher during winter versus other seasons (*P* < 0.001). In three hospitals in Shizuoka located in Japan in 1970–1999 (Sumiyoshi et al. 2002). excess admissions due to aortic aneurysm dissection were recorded between December and March. In the province of Ontario, Canada in 1988–1997 (Upshur et al. 2000). however, seasonal variations of aortic aneurysm and dissection admissions were not found. In a single hospital located in Greece in 1991–1995 and in a single hospital located in Italy in 1982–1994 (Kakkos et al. 1997; Manfredini et al. 1997). more admissions were also found to occur in spring and late autumn. Based on the data from the International Registry of Acute Aortic Dissection that was lead by an American team, researchers observed a winter peak across climates (Mehta et al. 2005). Consistent with the findings from the present study, the weather as a whole has a role in aortic aneurysm admissions.

### Weather and diseases of veins

Literature specifically on the relationship of the weather and hospital admissions due to diseases of veins could have been more limited than other human diseases recorded in ICD (including diseases of arteries). In a single hospital in Patras located in Greece in 2007–2008 (Kakkos et al. 2010). peak admissions of thrombophlebitis were recorded in the summer and autumn months. Data from all hospitals across Scotland in 1981–2001 revealed that admissions peaked in late autumn and winter (Brown et al. 2009). Similar results were obtained in Sweden in 1987–2010 and in France in 1995–1998 (Zöller et al. 2013; Boulay et al. 2001). In the MASTER Registry in 25 Italian hospitals (in Italy) in 2002–2004, excess admissions were recorded in late autumn (Manfredini et al. 2009). while a study from a single hospital in 1998–2002 observed a peak in December (Gallerani et al. 2004). In a single hospital in Vienna located in Austria in 1996–2000 (Fink et al. 2002). thrombosis admissions peaked in winter. In the University Hospital of Geneva located in Switzerland in 1989–1994 (Bounameaux et al. 1996). the seasonal variations of thrombosis admissions were not significant. Admissions due to venous leg ulcer could have been observed to peak in the summer time. In a single clinic in Pszczyna, Poland (Simka 2010). higher frequency was found in the warmer days. Similar results were obtained in Essen, Germany previously (Klode et al. 2011). In the USA, deep venous thrombosis did not show seasonal variation in any region (Stein et al. 2004). This is close to what we have observed in Germany that the variation is minimal. In a single hospital located in Tunisia (Tahri et al. 2003). it was observed that mean temperature could have been correlated with oesophageal varices haemorrhage. Overall, these vein problems were mostly examined by season but not necessarily meteorological variables that have made research comparison rather difficult. Future research putting efforts looking into these would be warranted.

Of note, we compared literature on admissions, incidence or prevalence of human diseases but not death/mortality alone. This is because disease severity and treatment efficacy would be more important than the weather during this period as patients would have stayed in hospital until being discharged or died.

### Strengths and limitations

The current study has a few strengths. Firstly, our study is the first in assessing weather as biometeorological and hospital admissions due to arteries and veins by subtypes that have combined epidemiological, geographical and meteorological methods in Germany. Secondly, our data is limited to very recent years to ensure that we do not find significant statistical associations by chance alone through pooling decades of data. Thirdly, we have drawn clear study catchments across Germany to ensure that both medical data and meteorological data can be matched geographically to rule out the potential ecological bias (i.e., one weather station plus numerous hospitals from other sub-regions). To be specific, in the current analysis, we ensured that in each German state, there could be at least one weather station to provide valid weather data to be correlated with hospital admissions within the state. Since we analysed the situation in Germany as a whole but not by sub-region/state, we chose to produce two-way fractional-polynomial prediction plots (Royston et al. 1999) rather than performing spatial-temporal modelling. Such statistical modelling selection (while non-linear testing and fitting were preferred to linear testing and fitting such as time-series regression modelling) has already been discussed and used since the 1980s (Kalkstein and Davis 1989). albeit less commonly used in correlating the weather and diseases of arteries and veins without an indication of dose–response relationship. In the present study, it was also used to provide insight regarding the potential relationships between human physiological thermal sensation and subsequent medical outcomes.

There were still other limitations worthy of being noted. First, we were unable to link with other population surveys to have adjusted covariates on lifestyle. In other words, this present study is at an ecological scale but not individual. Therefore, no causation could be drawn. By examining the correlations between the weather as biometeorological and hospital admissions, it is to indicate how much additional medical and social resources, such as medical professional-time and hospital facilities (Shiue and Matzarakis 2011). might be anticipated across each German state and/or nationally. Second, due to the restriction of the German law, we were only able to examine at the state level but not within smaller geographic regions. Due to the lack of complete weather data in three German states, this has made our statistical analysis not perfectly complete at the national level. From the meteorological point of view, when investigating the weather effect, it would make scientific sense to generate climatic variables into single index since they interact with each other at the same time, and 1 °C in a cold climate would mean differently in a warm climate. Although there are other indexes such as Universal Thermal Climate Index (UTCI), the difference between UTCI and PET was not much (Shiue et al. 2014). On the other hand, although daylight and sunshine hours were previously found to be statistically associated with health outcomes, they are influencing the degree of air temperature. Therefore, they were not specifically included in the present study. Of note, however, we did include solar radiation. Third, we did not include air pollution data due in part to the fact that by adjusting for air pollution in the epidemiological and statistical modelling, the effects could be stronger and easily reach statistical significance (Sabetghadam and Ahmadi-Givi 2014). The other practical reason was that the level of air pollution in Germany has been low in the recent years (more details via http://www.umweltbundesamt.de/en/data/current-concentrations-of-air-pollutants-in-germany). Therefore, the effect from air pollution would be minimal. Fourth, clinically, the waiting time between making appointments and actual admissions, if not emergency, could vary from days to months depending on specialisation in Germany. However, such information was not available within the current limited dataset. Consequently, any lag effect of the weather was not available to be analysed in the present study. Fourth, although diseases of arteries and veins could be acute that would require the immediate medical attention, some cases in a chronic condition during Christmas might wait until New Year leading to a drop of cases recorded in the last half of December. Unfortunately, we were unable to identify these cases because we only had ‘month’ but not ‘date’ documented in the current limited data set. Taken together, future studies keeping the strengths and overcoming the limitations mentioned above with a continuous monitoring would be warranted.

### Research, practice and policy implications

In sum, more hospital admissions due to diseases of arteries and veins by subtypes were observed on days with lower PETs across Germany, although the causality cannot be confirmed due to the ecological study design in nature. However, nationally, how to prepare reallocation of medical and social resources in response to adapting to the change of weather, in particular at PET −10 to 10 °C during the unstable and cold climate state, would seem to be important. In facing the climate change in the next decades, continuous monitoring with research on the weather and its influence on health and well-being would be warranted. From the practice and policy perspectives, consideration of including public attitude and adaptation strategies in addition to rapid reallocation of medical resources to save human capital in this country should be encouraged.

## Electronic supplementary material

Below is the link to the electronic supplementary material.

Supplementary Fig. 1 The included weather stations in Germany (DOCX 681 kb)

Supplementary Fig. 2 Averaged PET by month and by day over 3 years in 2009–2011 (DOCX 42 kb)
