# Supplementary figures and images for: Hospital admissions due to diseases of arteries and veins peaked at physiological equivalent temperature −10 to 10 °C in Germany in 2009–2011
Source: Environ Sci Pollut Res Int. 2015 Dec 3;23:6159–67. doi: 10.1007/s11356-015-5791-x (PMC4820476; doi:10.1007/s11356-015-5791-x)

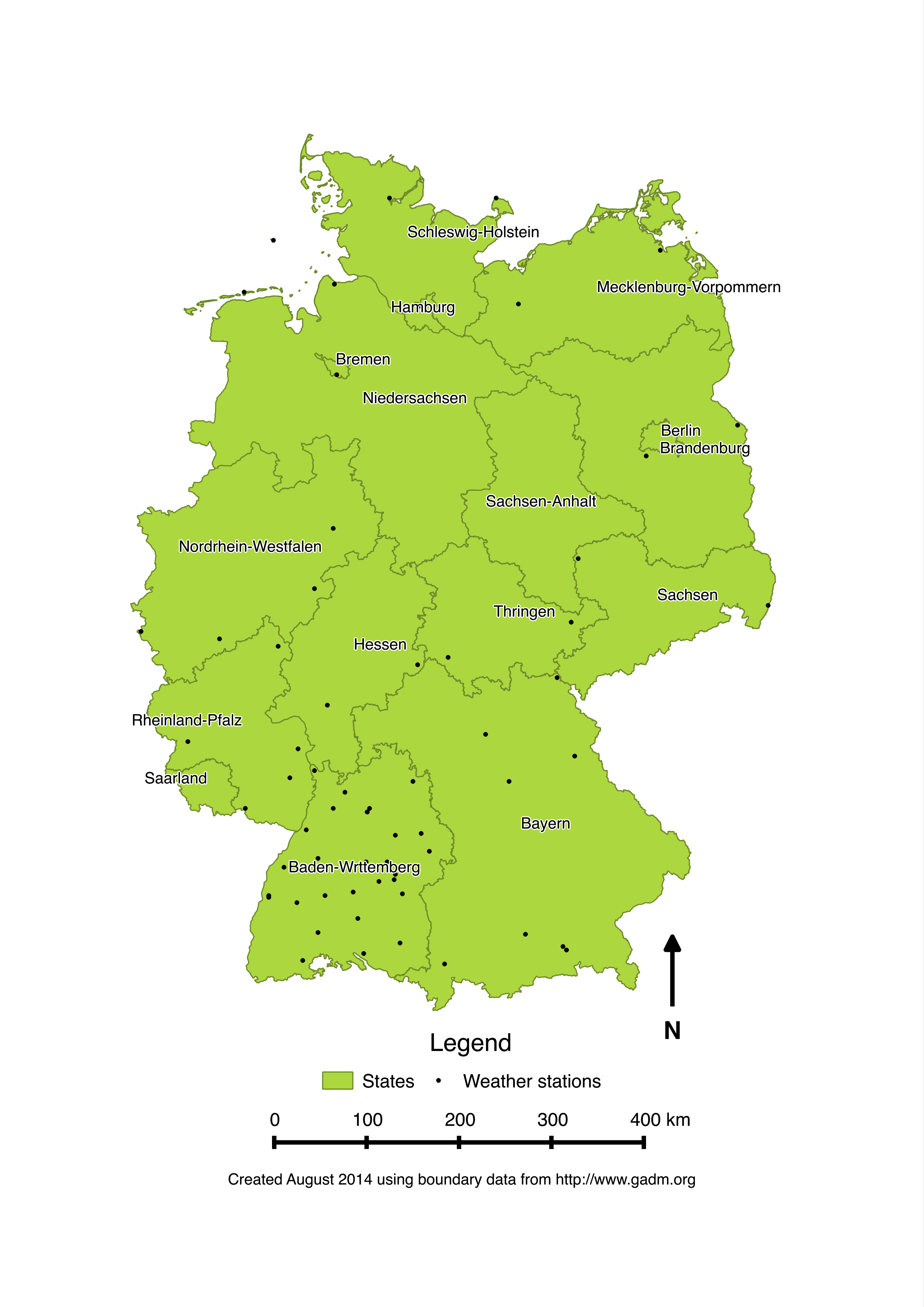

Supplement: Supplementary file 1 — The included weather stations in Germany (DOCX 681 kb) [file 11356_2015_5791_MOESM1_ESM.docx]

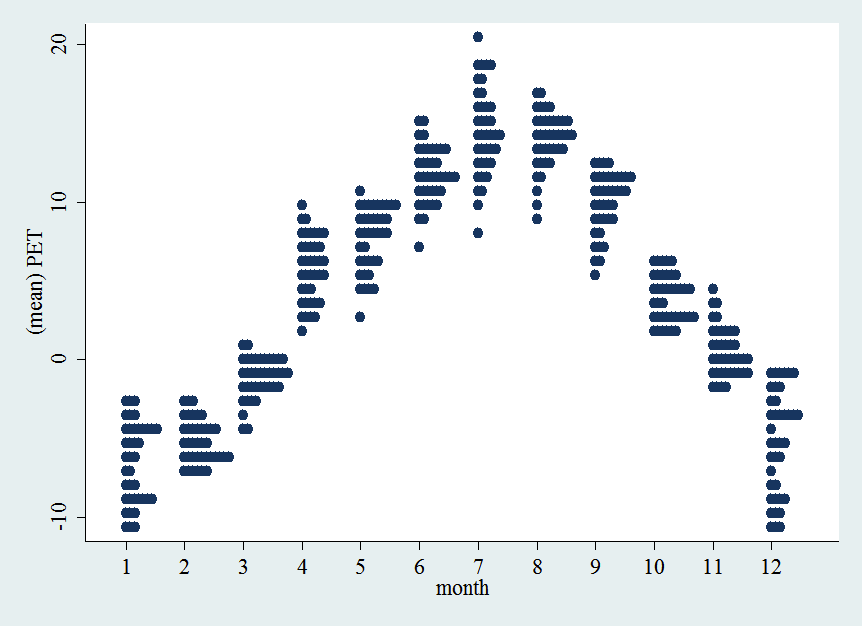

Supplement: Supplementary file 2 — Averaged PET by month and by day over 3 years in 2009–2011 (DOCX 42 kb) [file 11356_2015_5791_MOESM2_ESM.docx]
